# Targeting Platelet GPVI Plus rt-PA Administration but Not α2β1-Mediated Collagen Binding Protects against Ischemic Brain Damage in Mice

**DOI:** 10.3390/ijms20082019

**Published:** 2019-04-24

**Authors:** Michael K. Schuhmann, Peter Kraft, Michael Bieber, Alexander M. Kollikowski, Harald Schulze, Bernhard Nieswandt, Mirko Pham, David Stegner, Guido Stoll

**Affiliations:** 1 Department of Neurology, University Hospital Würzburg, 97080 Würzburg, Germany; peter.kraft@klinikum-msp.de (P.K.); bieber_m@ukw.de (M.B.); stoll_g@ukw.de (G.S.); 2 Department of Neurology, Klinikum Main-Spessart, 97816 Lohr, Germany; 3 Department of Neuroradiology, University Hospital Würzburg, 97080 Würzburg, Germany; kollikowsk_a@ukw.de (A.M.K.); pham_m@ukw.de (M.P.); 4 Institute of Experimental Biomedicine-Department I, University Hospital Würzburg, 97080 Würzburg, Germany; harald.schulze@uni-wuerzburg.de (H.S.); bernhard.nieswandt@virchow.uni-wuerzburg.de (B.N.); stegner@virchow.uni-wuerzburg.de (D.S.); 5 Rudolf Virchow Center, DFG Research Center for Experimental Biomedicine, University of Würzburg, 97080 Würzburg, Germany

**Keywords:** ischemic stroke, integrin α2, glycoprotein VI, recombinant tissue-type plasminogen activator, intracranial bleeding, transient middle cerebral artery occlusion

## Abstract

Platelet collagen interactions at sites of vascular injuries predominantly involve glycoprotein VI (GPVI) and the integrin α2β1. Both proteins are primarily expressed on platelets and megakaryocytes whereas GPVI expression is also shown on endothelial and integrin α2β1 expression on epithelial cells. We recently showed that depletion of GPVI improves stroke outcome without increasing the risk of cerebral hemorrhage. Genetic variants associated with higher platelet surface integrin α2 (ITGA2) receptor levels have frequently been found to correlate with an increased risk of ischemic stroke in patients. However until now, no preclinical stroke study has addressed whether platelet integrin α2β1 contributes to the pathophysiology of ischemia/reperfusion (I/R) injury. Focal cerebral ischemia was induced in C57BL/6 and *Itga2^−/−^* mice by a 60 min transient middle cerebral artery occlusion (tMCAO). Additionally, wild-type animals were pretreated with anti-GPVI antibody (JAQ1) or Fab fragments of a function blocking antibody against integrin α2β1 (LEN/B). In anti-GPVI treated animals, intravenous (IV) recombinant tissue plasminogen activator (rt-PA) treatment was applied immediately prior to reperfusion. Stroke outcome, including infarct size and neurological scoring was determined on day 1 after tMCAO. We demonstrate that targeting the integrin α2β1 (pharmacologic; genetic) did neither reduce stroke size nor improve functional outcome on day 1 after tMCAO. In contrast, depletion of platelet GPVI prior to stroke was safe and effective, even when combined with rt-PA treatment. Our results underscore that GPVI, but not ITGA2, is a promising and safe target in the setting of ischemic stroke.

## 1. Introduction

Ischemic stroke (IS) is a major cause of death and invalidity in the aging population with limited treatment options [1,2]. The primary therapeutic goal is the restoration of blood flow by either pharmacological thrombolysis and/or mechanical thrombectomy. However, recanalization by either technique, despite their undisputed potential to induce a favorable clinical outcome, remains futile in a significant number of treated patients. In addition, cerebral hemorrhage may ensue technically successful recanalization. The pathophysiological causes of futile recanalization and of postischemic cerebral hemorrhage after recanalization remain poorly understood. In this context ischemia/reperfusion (I/R), the potentially harmful aspect of blood flow returning to organs after transient ischemia, is arguably one of the most important concepts, which, in particular, applies to the ischemic brain [3]. Platelet activation plays a key role in the pathomechanism underlying IS and I/R injury. Administration of the conventional anti-platelet agent acetylsalicylic acid (ASA) in conjunction with intravenous (IV) recombinant tissue plasminogen activator (rt-PA) thrombolysis led to unacceptable bleeding complications [4]. To improve the outcome in acute ischemic stroke, novel anti-thrombotic/anti-platelet treatment options are urgently needed.

After the initial recruitment of platelets through interactions between the glycoprotein (GP) Ib-V-IX complex and collagen-bound von Willebrand factor (VWF) [5], platelets are immobilized via direct platelet-collagen binding. Thereby, glycoprotein VI (GPVI) functions as the central activating collagen receptor [6,7] which further triggers cellular activation, thus enabling integrin-mediated platelet adhesion and, eventually, aggregation. Of note, recently, other ligands of GPVI, namely fibronectin, vitronectin, laminin, adiponectin, EMMPRIN and fibrin [8] have been identified.

Following platelet activation, integrins undergo conformational changes to mediate firm adhesion via GPIIb/IIIa (αIIbβ3) and vWF or α2β1 (GPIa/IIa) and collagen [6,9]. Utilizing integrin α2-deficient (*Itga2^−/−^*) mice as well as an anti-α2β1 antibody (LEN/B Fab-fragment) treatment to analyze the relevance of this integrin for platelet function during hemostatic and thrombotic conditions revealed that α2β1 plays a significant, but only supportive role in platelet adhesion to collagen compared to the major activating collagen receptor GPVI [6,10,11,12]. We and others have previously shown that targeting platelet-collagen interactions during cerebral ischemia, namely antibody mediated depletion of platelet GPVI or blockade of platelet binding to the vessel walls by Revacept, a GPVI-Fc fusion protein competitively inhibiting GPVI, reduced microvascular thrombosis and infarct volumes after a transient middle cerebral artery occlusion (tMCAO) in mice [13,14,15].

With these considerations in mind, the aim of our study was to investigate the pathogenetic relevance of α2β1-mediated collagen binding during the acute phase of IS development using two experimental approaches (LEN/B antibody treatment; *Itga2^−/−^* mice). The occurrence of hemorrhagic transformation and intracranial hemorrhages in acute IS is potentially at least in part a consequence of rt-PA induced alterations at the blood brain barrier [16,17,18]. Therefore, in addition we asked whether targeting the collagen receptor GPVI is still safe with regard to bleeding complications when it is performed in conjunction with rt-PA treatment after tMCAO.

## 2. Results

### 2.1. Targeting α2β1-Integrin Does Not Improve Outcome after tMCAO

**Figure 1:**
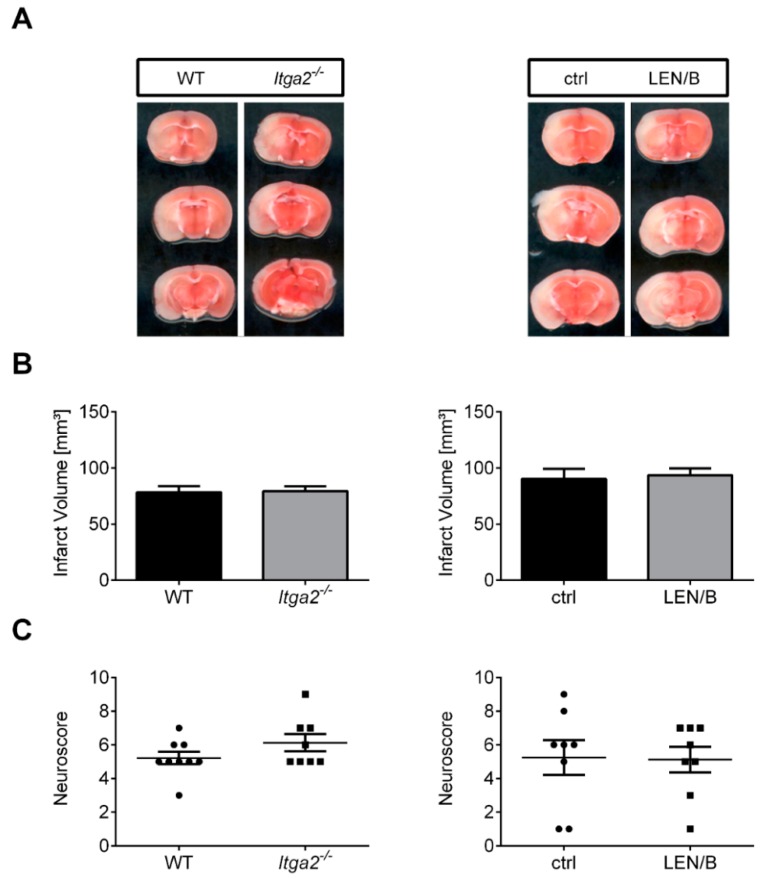
Therapeutic blockade or genetic deficiency of the collagen-binding integrin α2β1 does not alter stroke outcome in a transient middle cerebral artery occlusion (tMCAO) model. (**A**) Representative 2,3,5-triphenyltetrazolium chloride stains of three corresponding brain sections of a wild-type (WT) mouse in comparison to an *Itga2^−/−^* mouse at day 1 after tMCAO (**left**) and a vehicle-treated C57BL/6 mouse (ctrl) compared to a C57BL/6 mouse treated with a specific α2β1-antigen binding fragment (LEN/B) 1 h before 60 min tMCAO at day 1 after stroke (**right**). (**B**) Infarct volumes are similar between the two experimental (pharmacologic; transgenic mice) groups (WT, *n* = 9; *Itga2^−/−^*/ctrl/LEN/B, *n* = 8/group; unpaired, two-tailed Student’s *t*-test). (**C**) Genetic (**left**) or pharmacologic (**right**), α2β1 inhibition does not improve functional outcome on day 1 after tMCAO as assessed by the Neuroscore (Mann–Whitney test).

First, we assessed if pharmacologic blockade of α2β1 (LEN/B treatment) before tMCAO influences stroke development in wild-type (WT) mice. Stroke volumes (ctrl: 90.3 ± 9.0 mm^3^; LEN/B: 93.6 ± 6.1 mm^3^; *p* > 0.05; Figure 1A,B, right panel) and functional outcomes as assessed by the Neuroscore (median with (25% and 75%) percentile: ctrl: 6.0 (2.0, 7.5); LEN/B: 5.5 (3.5, 7.0); *p* > 0.05) on day 1 did not significantly differ (Figure 1C, right panel). In addition, to exclude that the LEN/B antibody treatment influences outcome measures independent of its α2β1-blocking effect, we analyzed *Itga2^−/−^* mice. Again, there was no significant difference in infarct volumes (WT: 78.4 ± 5.5 mm^3^; *Itga2^−/−^*: 79.4 ± 4.4 mm^3^; *p* > 0.05) (Figure 1A,B, left panel) as well as functional outcomes (median with (25% and 75%) percentile: WT: 5.0 (5.0, 6.0); *Itga2^−/−^*: 5.5 (5.0, 7.0); *p* > 0.05) (Figure 1C, left panel) when comparing *Itga2^−/−^* with WT control mice at day 1 after stroke.

### 2.2. Blocking the Collagen Receptor GPVI Together with IV rt-PA Treatment after tMCAO Is Safe and Effective

**Figure 2:**
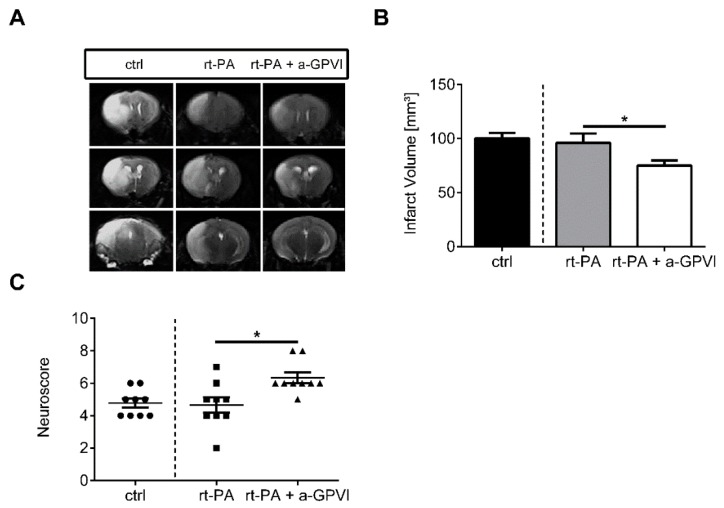
Blocking of glycoprotein VI (GPVI) mediated collagen binding together with rt-PA treatment improves stroke outcome in a transient middle cerebral artery occlusion (tMCAO) model. (**A**) Representative serial coronal T2-weighted turbo spin echo MR images of vehicle-treated wild-type mice, mice treated with rt-PA or both rt-PA and JAQ1 (α-GPVI) 24 h after induction of tMCAO. Ischemic infarctions appear hyperintense (bright). (**B**) Magnetic resonance imaging (MRI)-based infarct volumetry (ctrl/rt-PA/rt-PA + a-GPVI, *n* = 9/group; unpaired, two-tailed Student’s *t*-test). (**C**) Analysis of the functional outcome on day 1 after tMCAO as assessed by the Neuroscore (Mann–Whitney test). * *p* < 0.05.

In line with our previous studies [14,15], GPVI depletion by anti-GPVI antibody (JAQ1) treatment significantly reduced infarct volumes at day 1 after tMCAO (rt-PA: 96.1 ± 8.6 mm^3^; rt-PA + a-GPVI: 75.1 ± 4.7 mm^3^; *p* < 0.05; Figure 2A,B) when combined with pharmacological IV rt-PA treatment. Analysis of functional outcome revealed that reduced stroke size in the anti-GPVI treated mice also translated into a better Neuroscore (median with (25% and 75%) percentile: rt-PA: 5.0 (4.0, 5.5); rt-PA + a-GPVI: 5.0 (6.0, 7.0); *p* < 0.05; Figure 2C). We here conducted magnetic resonance (MR) imaging partly because in addition to the quantification of infarct volumes, this enabled us to quantify the occurrence of cerebral hemorrhages in parallel. Importantly, quantification of intracerebral hemorrhage (ICH) iron-sensitive susceptibility weighted imaging (SWI) sequence MR images at day 1 after tMCAO according to a 0–2 scoring system [16], revealed no increase in the occurrence of cerebral hemorrhages when blocking platelet GPVI during rt-PA treatment (Table 1).

## 3. Discussion

In the present study, pharmacologic blockade of the platelet integrin α2β1 and *Itga2^−/−^* mice was tested independently. Experiments revealed that platelet activation, as well as aggregation in the ischemic brain, does not depend on α2β1-collagen interactions and stroke outcome was comparable. Therefore, this adhesion receptor plays a minor role in acute IS compared to the major activating collagen receptor GPVI [14,15,19]. This is in good agreement with previous in vitro and in vivo studies that found the integrin α2β1 to be of minor relevance in models of arterial thrombosis [10] when compared to GPVI, albeit it also significantly contributes to the adhesion of platelets to collagen [6,12]. A possible explanation for the superiority of targeting GPVI might be due to interactions with ligands other than collagen [8]. Among these, a role of GPVI as a functional platelet receptor for polymerized fibrin has recently been identified. By this, GPVI is critically involved in the amplification of thrombin generation and platelet recruitment to clots [20]. Thrombin formation has been shown to be involved in the induction of inflammation, thrombus formation and infarct development in experimental ischemic stroke [21]. Importantly, when combining GPVI-deficiency together with rt-PA treatment after tMCAO, an experimental setting that closely resembles the current clinical treatment situation where patients with a severe ischemic stroke (caused by large-vessel-occlusion) are treated by mechanical recanalization with the concomitant pharmacological support of IV administered rt-PA, the lack of GPVI was still safe and effective. With respect to the devastating results (bleeding complications) when blocking thromboxane release by ASA [4] or blocking platelet aggregation by GPIIb/IIIa inhibitors [14,15,22,23], our data provide further translational evidence that targeting platelet GPVI is an effective and safe treatment in the setting of acute IS. Despite the strengths of our investigation, additional studies are needed to finally evaluate the safety profile of depleting GPVI together with rt-PA treatment. In the present study rt-PA was infused at 60 min after tMCAO which may be too early to see the bleeding, but the risk of bleeding may dramatically increase at later time points after stroke. Additionally, an embolic stroke model is required in order to assess thrombolysis by rt-PA.

Taken together our study provides evidence that targeting platelet GPVI but not α2β1 integrin protects mice from ischemic stroke in the acute phase after tMCAO.

## 4. Materials and Methods

### 4.1. Animals and Ethics

We used 10–12-week old male C57Bl/6 and *Itga2^−/−^* mice [11] (backcrossed onto C57Bl/6 > 12 times) throughout this study. All animal experiments were approved by local state authorities (Regierung von Unterfranken) and performed in accordance with Animal Research: Reporting In Vivo Experiments (ARRIVE) guidelines (http://www.nc3rs.org/ARRIVE). All applicable international, national and/or institutional guidelines for the care and use of animals were followed.

### 4.2. Animal Treatment

To inhibit platelet α2β1, mice received 5 mg/kg of anti-α2β1 antibody (LEN/B-Fab fragment) 1 h before tMCAO [12]. To deplete GPVI, mice were given 100 μg JAQ1 intraperitoneally [24], five days prior to tMCAO. After 60 min of tMCAO, 10 mg/kg of human rt-PA (Actilyse; Boehringer-Ingelheim) was injected as an intravenous bolus directly before reperfusion [18].

### 4.3. Sample Size Calculation

Based on the results of our previous studies using anti-GPVI treated mice, we expected a reduction of infarct volumes of ~32% [14]. Assuming a standard deviation of 20% to the respective mean values, a group size of ≥7 mice was necessary to show this effect with a power of 0.8 and a probability of a type I error of <0.5 (GraphPad StatMate2.0 software).

### 4.4. tMCAO

Focal cerebral ischemia was induced by 60 min tMCAO, exactly as described previously [14]. To calculate edema-corrected infarct volumes, 2,3,5-triphenyltetrazolium chloride stained brain slices were used. Neurologic function was analyzed calculating a Neuroscore (score 0–10) based on the direct sum of the Grip test (score 0–5) and the inverted Bederson score (score 0–5) [18,25,26]. 

### 4.5. Exclusion Criteria

Mice were excluded from endpoint analyses due to: (1) death before the predefined experimental endpoint; (2) Bederson score = 0 (24 h after tMCAO); (3) Operation time >10 min.

### 4.6. Magnetic Resonance Imaging

To analyze infarct size and to scan for possible intracerebral bleeding, stroke assessment in rt-PA/anti-GPVI treated mice was performed by serial magnetic resonance imaging (MRI) at 3.0 T (MAGNETOM Trio, SIEMENS, Erlangen, Germany) on day 1 after tMCAO. The image protocol comprised a coronal multi-slice TSE (TR/TE (Repetition Time/Echo Time) = 2100/113 ms, 18 slices, BW (Bandwidth) = 120 Hz/px, In-plane-resolution: 0.17 × 0.17 × 1.0 mm^3^) as well as a 3D CISS (TR/TE = 11.57/4.87 ms, BW = 130 Hz/px, In-plane-resolution: 0.25 × 0.25 × 0.5 mm^3^, FA (Flip Angle) = 70°) for T2-weighted imaging. Additionally, a 3D GRE SWI (TR/TE = 38 ms, BW = 140 Hz/px, In-plane-resolution: 0.16 × 0.16 × 0.16 mm^3^, FA = 20°) for susceptibility weighted imaging was acquired. MRIs were visually assessed by researchers blinded to the prior treatment with respect to infarct morphology and, in particular, the occurrence of intracranial hemorrhage [14].

### 4.7. Evaluation of Bleeding Severity (SWI Score)

The susceptibility weighted imaging (SWI) score was applied to quantify bleeding severity after tMCAO, exactly as described previously [18]. The scoring is as follows: score 0, no bleeding was detectable; score 1, one SWI lesion suggestive for intracerebral hemorrhage; score 2, two or more different SWI lesions were visible.

### 4.8. Statistical Analysis

For statistical analysis, the GraphPad Prism 5.0 software package (GraphPad Software, La Jolla, CA) was used. Results are given as mean ± standard error of the mean except for the Neuroscore, which is expressed as ordinal values. Data were tested for Gaussian distribution with the D’Agostino and Pearson omnibus normality test and then analyzed by unpaired, two-tailed Student’s *t*-test. Scores addressing the functional outcome were compared using the Mann–Whitney *U*-test. *p* < 0.05 was considered statistically significant.

## Figures and Tables

**Table 1:** Susceptibility weighted imaging (SWI) Score of rt-PA Treated Mice at Day1 after tMCAO.

SWI Score	0	1	2
**rt-PA**	6/9	2/9	1/9
**rt-PA + a-GPVI**	7/9	2/9	0/9
